# The Use of Digital Devices to Monitor Physical Behavior in Motor Neuron Disease: Systematic Review

**DOI:** 10.2196/68479

**Published:** 2025-04-17

**Authors:** Lucy Samantha Musson, Nina Mitic, Victoria Leigh-Valero, Gladys Onambele-Pearson, Liam Knox, Frederik J Steyn, Cory J Holdom, Taylor JM Dick, Ruben PA van Eijk, Jordi WJ van Unnik, Lianne CM Botman, Emily Beswick, Deirdre Murray, Alys Griffiths, Christopher McDermott, Esther Hobson, Amina Chaouch, Emma Hodson-Tole

**Affiliations:** 1 Sheffield Institute for Translational Neuroscience Division of Neuroscience University of Sheffield Sheffield United Kingdom; 2 Department of Life Sciences Manchester Metropolitan University Manchester United Kingdom; 3 University of Manchester Manchester United Kingdom; 4 Department of Sport and Exercise Sciences Manchester Metropolitan University Manchester United Kingdom; 5 School of Biomedical Sciences University of Queensland St Lucia, Queensland Australia; 6 Department of Neurology Royal Brisbane and Women’s Hospital Herston, Queensland Australia; 7 Australian Institute for Bioengineering and Nanotechnology University of Queensland St Lucia, Queensland Australia; 8 Department of Neurology UMC Utrecht Brain Center University Medical Center Utrecht Utrecht The Netherlands; 9 Biostatistics & Research Support Julius Center for Health Sciences and Primary Care University Medical Center Utrecht Utrecht The Netherlands; 10 Academic Unit of Neurology School of Medicine Trinity College Dublin Dublin Ireland; 11 Sheffield Teaching Hospitals NHS Foundation Trust Sheffield United Kingdom; 12 Manchester Centre of Clinical Neurosciences Salford Royal NHS Foundation Trust Salford United Kingdom

**Keywords:** motor neuron disease, amyotrophic lateral sclerosis, physical behavior, digital devices, remote monitoring, wearable technology

## Abstract

**Background:**

Motor neuron disease (MND) is a progressive and incurable neurodegenerative disease. The Amyotrophic Lateral Sclerosis Functional Rating Scale-Revised (ALSFRS-R) is the primary clinical tool for assessing disease severity and progression in MND. However, despite its widespread use, it does not adequately capture the extent of physical function decline. There is an urgent need for sensitive measures of disease progression that can be used to robustly evaluate new treatments. Measures of physical function derived from digital devices are beginning to be used to assess disease progression. There is value in establishing a consensus approach to standardizing the use of such devices.

**Objective:**

We aimed to explore how digital devices are being used to quantify free-living physical behavior in MND. We evaluated the feasibility and assessed the implications for monitoring physical behavior for future clinical trials and clinical practice.

**Methods:**

Systematic searches of 4 databases were performed in October 2023 and June 2024. Peer-reviewed English-language articles (including preprints) that examined how people living with MND used digital devices to assess their free-living physical behavior were included. Study reporting quality was assessed using a 22-item checklist (maximum possible score=44 points).

**Results:**

In total, 12 articles met the inclusion criteria for data extraction. All studies were longitudinal and observational in design, but data collection, analysis, and reporting protocols varied. Quality assessment scores ranged between 19 and 40 points. Sample sizes ranged between 10 and 376 people living with MND at baseline, declining over the course of the study. Most studies used an accelerometer device worn on the wrist, chest, hip, or ankle. Participants were typically asked to continuously wear devices for 1 to 8 days at 1- to 4-month intervals, with studies running for 12 weeks to 24 months. Some studies asked participants to wear the device continuously for the full duration. Studies derived traditional end points focusing on duration, intensity, and frequency of physical activity or nontraditional end points focusing on features of an individual’s movement patterns. The correlation coefficients (*r*) between physical behavior end points and ALSFRS-R ranged from 0.31 to 0.78. Greater monitoring frequencies and improved end point sensitivity were shown to provide smaller sample size requirements and shorter durations for hypothetical clinical trials. People living with MND found using devices acceptable and reported a low burden. Adherence assessed in 8 (67%) studies was good, ranging from approximately 86% to 96%, with differences evident between wear locations. The perspectives of other end users and implications on clinical practice were not explored.

**Conclusions:**

Remote monitoring of free-living physical behavior in MND is in its infancy but has the potential to quantify physical function. It is essential to develop a consensus statement, working toward agreed and standardized methods for data collection, analysis, and reporting.

## Introduction

### Background

Motor neuron disease (MND), a group of progressive neurodegenerative disorders that includes amyotrophic lateral sclerosis (ALS), is characterized by loss of motor neurons in the brain, brainstem, and the spinal cord [1]. Most people living with MND experience progressive weakness and wasting of their muscles with a life expectancy of only 2 to 3 years following symptom onset [2]. There is no cure for MND, and care is based on providing symptomatic support through multidisciplinary teams. These teams must carefully monitor physical function, nutritional status, respiratory function, cognition, and well-being to inform clinical decision-making and provide timely and effective support. People living with MND are usually reviewed every 3 months [3]. However, disease progression is variable, with some people living with MND requiring more frequent monitoring, while others need less frequent input due to slower disease progression.

The most used functional measure of disease severity and progression in MND is the Amyotrophic Lateral Sclerosis Functional Rating Scale-Revised (ALSFRS-R) [4]. This questionnaire measures changes in function across 4 domains (bulbar, fine motor, gross motor, and respiratory) in the context of completing activities of daily living [4]. The ALSFRS-R is predictive of survival and is commonly used as a primary end point in clinical trials [5,6]. However, it has limitations. It has been shown that the total score of the ALSFRS-R is multidimensional and does not accurately capture the heterogeneity of people living with MND. This means that 2 individuals can have the same total ALSFRS-R score but have different disease severity, experience different symptoms, and have different prognoses [7-9]. This can result in under or overestimating treatment effects in clinical trials [10]. Moreover, the ALSFRS-R is not particularly sensitive to disease progression over durations <12 months [9]. Thus, there is a need for more objective and sensitive ways of characterizing disease progression in MND. To do this, it is likely that bespoke tools that provide sensitive assessment across the 4 disease domains must be developed. In this review, we focus on the currently available tools to assess physical behavior in people living with MND.

Digitally-derived measures of physical behavior have been identified as potential markers of disease onset, progression, and response to treatment in neurodegenerative diseases. A recent systematic review of the literature revealed 17 reports of activity monitoring in people living with Parkinson disease and highlighted their value and application in well-designed clinical trials [11].

There has also been growing interest in how digital technologies can be used to monitor symptoms in people living with MND [12,13] and several different devices have been used in research to evaluate motor symptoms associated with MND in people living with MND. For example, Geronimo et al [14] found that inertial sensors can collect gait data as a biomarker that is sensitive to changes in physical function in people living with MND. This study explored the use of digital devices in the clinic where patients were guided by a therapist. A review found that studies have also explored the potential for these devices to monitor a person’s free-living behavior, which allows observation of typical behavior in everyday life [15]. For this, one technology that seems particularly promising is wearable triaxial accelerometer devices. These small devices can be worn unobtrusively (eg, on the wrist like a watch or on the waist on a belt) and detect accelerations of the body in 3 orthogonal planes. They enable noninvasive monitoring of people undertaking their free-living, habitual daily activities outside a clinical or research environment. This includes being active, sedentary, and sleeping, which, when taken together, can be considered a person’s physical behavior pattern [16].

Building on the review by Beswick et al [15], it is timely to investigate current knowledge of physical behavior patterns in MND and the methods by which this knowledge is being accrued so that standards for best practice can be identified and shared. This will not only highlight the potential value of remote monitoring of physical behavior in people living with MND, but may also offer a stepping stone for applying the knowledge to other progressive diseases.

### Objectives

Therefore, this systematic review aimed to (1) explore how digital devices are being used to quantify free-living physical behavior in people living with MND, (2) evaluate the feasibility of using these devices for objectively delineating the physical impact of MND, and (3) assess the implications of physical behavior monitoring for clinical trials design and clinical practice.

## Methods

### Search Strategy

A systematic review of scientific literature (written in English) was conducted in October 2023 using 4 databases as follows: Europe PMC (October 11, 2023), SCOPUS (October 11, 2023), Web of Science (October 11, 2023), and IEEE Xplore (October 12, 2023). The included articles were not restricted by the date of publication. The search was performed in line with the current PRISMA (Preferred Reporting Items for Systematic Reviews and Meta-Analyses) statement [17] (Multimedia Appendix 1). To ensure the work published here was as current as possible, an updated search of each database was conducted as follows: Europe PMC (June 20, 2024), SCOPUS (June 20, 2024), Web of Science (June 20, 2024), and IEEE Xplore (June 20, 2024). Here, the review period was limited to the years 2023 to 2024, with the aim of identifying and including any study published between the date of the initial search and final manuscript preparation.

The following search strategy was used in each database: “(MND or ALS or motor neurone disease or motor neuron disease or amyotrophic lateral sclerosis) AND (physical activity or exercise or physical behaviour or sedentary behaviour or mobility) AND (remote monitoring or sensors or digital technology or accelerometer* or actigraphy or GPS or wearable technology or objective monitoring or wearable devices).”

### Screening for Eligibility

The full inclusion and exclusion criteria are presented in Textbox 1. All references were imported to the Rayyan (Rayyan Systems, Inc) web tool [18] for initial screening. A total of 336 records were identified during the initial search of databases. Following the removal of duplicates (63/336, 18.8%), articles were screened to assess eligibility. Initial screening was completed by NM. Studies were initially screened by title alongside the inclusion and exclusion criteria (ie, titles indicating a systematic review, investigation of healthy populations, or not specific to remote monitoring of physical behavior were excluded). This was followed by abstract and full-text screening. Forward and backward reference chaining from eligible articles was completed to identify other studies not captured by the search. A total of 12 articles met the criteria for data extraction.

**Study inclusion and exclusion criteria.**

**Inclusion criteria**

- Participants: study population includes people living with motor neuron disease
- Design: any other design not specified in the exclusion criteria
- Intervention of interest: use of remote monitoring devices to assess physical behavior
- Outcome of interest: remote monitoring of physical behavior in free-living conditions
- Setting: free-living environment, home or domiciliary monitoring, and remote monitoring
- Type of publication: peer-reviewed journal articles and preprint articles subject to secondary review
- Date of publication: no restriction
- Language of publication: English

**Exclusion criteria**

- Participants: participant with other neurological conditions
- Design: animal studies, ongoing trials, systematic reviews, and meta-analysis
- Intervention of interest: devices used for rehabilitation purposes (such as orthoses) and devices measuring any other parameters that are not physical behavior
- Outcome of interest: gait analysis–gait-specific parameters focused on identifying pathological gait patterns in the clinical environment and monitoring of prescribed exercises or set movement tasks
- Setting: face-to-face monitoring in clinical environment
- Type of publication: any other publication type (eg, conference abstract and book chapters) and preprints that are now published
- Language of publication: non-English

### Data Extraction

A data extraction tool was created by NM with a focus on extracting information relevant to the study aims (Multimedia Appendix 2). Two researchers (NM and LSM) independently extracted information from the identified studies. Once extracted, these authors compared results for agreement, with disputes resolved by a third reviewer (EHT).

### Quality Assessment

Despite the overarching observational nature of the studies, there was substantial heterogeneity in study design between them. Consequently, a decision was made to assess the reporting quality of the studies to inform future research in this area, and in doing so, support the evaluation of good practice in use and reporting of free-living physical behavior in MND. An a priori decision was made to include all eligible studies in the review regardless of their quality, due to the infancy of the research area.

The reporting quality of studies was assessed using the STROBE (Strengthening the Reporting of Observational Studies in Epidemiology) statement [19]. The STROBE guidelines provide researchers with a checklist of 22 items required for good reporting of observational studies [19]. In this study, an article was awarded 2 points for each item that was addressed, 1 point for each item deemed to be partially reported but required further information, and 0 when no information was provided. LSM and NM independently assessed each article using a Microsoft Excel spreadsheet. LSM and NM then met to confirm and resolve discrepancies.

### Data Collation

Two pairs of articles had identical methods, effectively reporting different aspects of the same study. In both cases, the first article of the pair [20,21] focused on the description of the method, while the second [22,23] summarized methods and focused on the main research findings. Therefore, when assessing the methods of data collection, the pairs were considered as 1 study (ie, the total number of studies for data collection was 10). When assessing study findings, each study in the pair was considered separately, so that 12 articles were included in the analysis of findings. If several studies used the same MND population, the participant data were taken from the study publishing findings (rather than articles focusing on methodology or feasibility) or the study that was published first. Several studies used data collected on the same participant population. Due to heterogeneity between the physical behavior end points used in these studies, we deemed it inappropriate to perform complete statistical analysis of findings; therefore, we focused on the narrative review of evidence, descriptive statistics of participant characteristics, and outcome measures used in the studies.

## Results

### Overview

**Figure 1:**
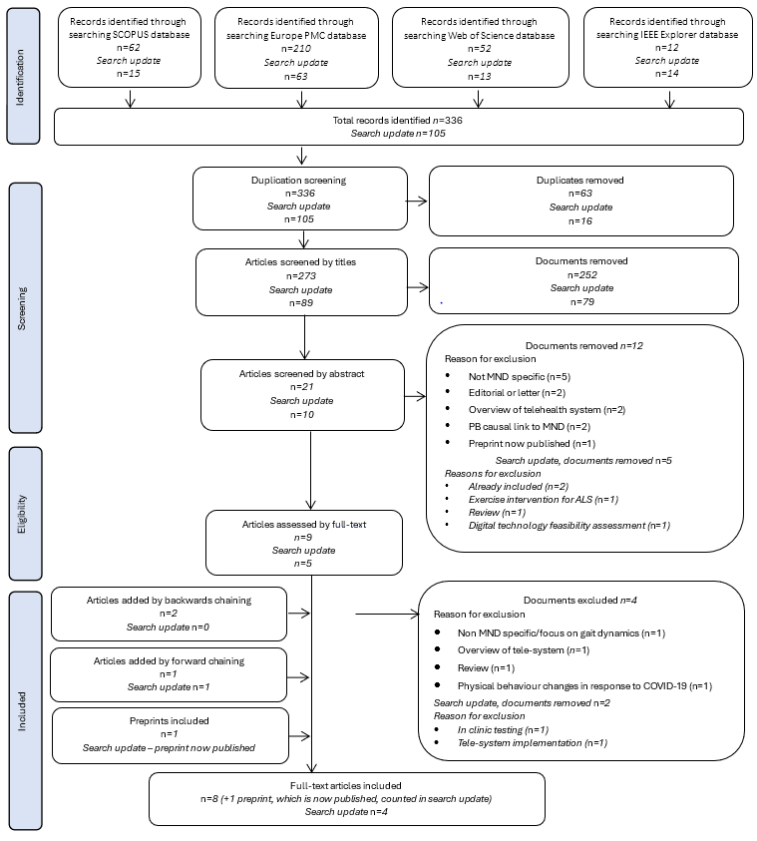
PRISMA (Preferred Reporting Items for Systematic Reviews and Meta-Analyses) flowchart of the search process, which includes the initial search in October 2023 and search update completed in June 2024 (date limit: 2023-2024). The number of articles at each stage was reported separately for each search. ALS: amyotrophic lateral sclerosis; MND: motor neuron disease.

Following the removal of duplicates and the full screening process, 12 articles published between 2019 and 2024 were included in the review [20-31]. Figure 1 provides a flowchart of the complete search process. Studies were conducted in 5 countries: Australia, Netherlands, Scotland, United Kingdom, and United States. We noted 3 overarching aims of investigation as follows: (1) validating remote monitoring of physical behavior in MND and finding markers of disease progression; (2) investigating feasibility of remote monitoring; and (3) investigating sample size effects of physical behavior end points to inform clinical trial design.

### Reporting Quality

Overall, the scores from the reporting quality assessment ranged between 19 and 40 (maximum available points=44; Table 1). The only item that was fully addressed by every article (100%) was the limitations of the study. While all articles attempted to discuss generalizability to all people living with MND (eg, limb vs bulbar onset), the reviewers agreed that all the articles would have benefited from providing more details about this. The most frequently missed item was a description of any efforts to address potential sources of bias, with all 12 (100%) articles failing to address this. In total, 8 (67%) articles did not explain how the study size was determined, and 3 (25%) articles failed to provide the eligibility criteria of participants or the methods of participant selection. In total, 2 (17%) articles failed to describe their statistical methods, and 2 (17%) articles failed to define all outcomes, exposures, predictors, potential confounders, and effect modifiers.

**Table 1:** Scores from the reporting quality assessment for each item of the STROBE (Strengthening the Reporting of Observational Studies in Epidemiology) statement for all studies included in the systematic review.

		Study (by author)											
STROBE statement		van Eijk et al [24] (38 points)	Garcia-Gancedo et al [20] (31 points)	Karas et al [25] (36 points)	Kelly et al [22] (35 points)	Rutkove et al [21] (19 points)	Rutkove et al [23] (25 points)	Holdom et al [26] (36 points)	Johnson et al [27] (36 points)	Gupta et al [28] (27 points)	van Unnik et al [29] (40 points)	Beswick et al [30] (37 points)	Straczkiewicz et al [31] (37 points)
Title and abstract		2	2	1	2	2	2	2	1	1	2	2	1
**Introduction**													
	Background and rationale	1	2	2	2	1	1	2	2	1	2	2	2
	Objectives	1	2	2	2	2	1	1	2	1	1	2	2
**Methods**													
	Study design	2	2	1	2	2	2	2	2	1	2	2	1
	Setting	2	1	2	1	1	1	2	2	1	2	1	2
	Participants	2	2	2	2	0	0	1	2	0	2	2	2
	Variables	2	1	2	1	0	0	2	2	1	2	2	2
	Data sources and measurement	2	2	2	2	1	1	2	2	1	2	1	2
	Bias	0	0	0	0	0	0	0	0	0	0	0	0
	Study size	2	2	0	2	0	0	0	0	0	2	0	0
	Quantitative variables	2	0	2	1	1	1	2	2	2	2	2	2
	Statistical methods	2	0	2	1	0	1	1	2	1	2	1	2
**Results**													
	Participants	1	2	2	2	1	2	2	1	1	1	2	2
	Descriptive data	2	2	2	2	1	1	2	2	2	2	2	2
	Outcome data	2	1	2	2	0	1	2	2	2	2	2	2
	Main results	2	1	2	1	0	1	2	2	2	2	2	2
	Other analyses	2	1	2	1	0	1	2	2	2	2	2	2
**Discussion**													
	Key results	2	2	2	2	1	2	2	2	2	2	2	2
	Limitations	2	2	2	2	2	2	2	2	2	2	2	2
	Interpretation	2	1	1	2	1	2	2	2	2	2	2	2
	Generalizability	1	1	1	1	1	1	1	1	1	2	2	1
	Funding	2	2	2	2	2	2	2	1	1	2	2	2

### Data Collection

All 10 (100%) studies used a longitudinal observational study design; however, methods were very heterogeneous. There were 4 elements identified as important to data collection methods as follows: (1) participant characteristics, (2) follow-up, (3) device setup, and (4) other outcome measures, described in subsequent sections.

#### Participant Characteristics

A summary of participant characteristics is presented in Table 2. The participant sample size varied across studies (10-376 people) and decreased over longitudinal measurement time points due to loss to follow-up and progression of the disease. However, it was often unclear (or not described) how many participants were included at each measurement point. Overall, there were more male participants (523/762, 68.6%) in the MND population compared to female (239/762, 31.4%). The studies reported age as either mean (7/12, 58%) or median (2/12, 17%). The overall mean age of participants in the studies reporting the mean was 59.7 years, while the median was 58.8 years. In total, 3 (30%) studies [21,26,28] included healthy controls, but the sample size was much smaller (25-58 individuals) and was not age or sex matched to the clinical population with median age of 51 years and had more female (61/113, 54%) participants compared to male (52/113, 46%) participants. The reported means and medians of the baseline ALSFRS-R scores ranged from 31.4 to 41.6 points. MND subtype or ALS phenotype at onset were reported in 7 (58%) studies. The most common subtype of MND was ALS, which represented 50% (5/10 participants) [30], 93% (39/42 participants) [24], 93% (87/94 participants) [26] and 97% (94/97 participants) [29] of the study’s baseline population, while the most common phenotype at onset was upper limb, which represented 60% (15/25) of the baseline population [20]. Of the 7 (70%) studies that reported symptom duration at baseline, 2 (29%) predominantly included those who had symptoms for ≤18 months, although it should be noted these 2 (29%) reports used the same dataset [20,22]. In the other 5 (5/7, 71%) studies, participants had predominantly experienced symptoms for >20 months [24-26,28,29].

**Table 2:** Summary of participant characteristics.

Study	Sample size (baseline), n	Controls, n	Sex, n					Age (y), mean (SD)		Disease phenotype, n		Symptom duration at baseline (mo), median and range		ALSFRS-R^a^ total score at baseline, mean (SD)
				Male		Female								
van Eijk et al [24]	MND^b^: 42	—^c^	31		11		60 (12)		MND subtypes: ALS^d^: 39; progressive muscular atrophy: 3; and primary lateral sclerosis: 0		25 (7-218)		36 (8)	
Garcia-Gancedo et al [20]	ALS: 25	—	21		4		53.1 (9.93)		Phenotype at onset: upper limb: 15; lower limb: 6; upper limb and lower limb: 2; and bulbar=2		Reported in 22 participants as <18 months		41.6 (4.98)	
Kelly et al [22]	ALS: 25	—	21		4		53.1 (9.93)		Phenotype at onset: upper limb: 15; lower limb: 6; upper limb and lower limb: 2; and bulbar: 2		<3 months: n=3; 3-6 months: n=8; 6-12 months: n=9; 1 year-18 months: n=2; and missing data: n=3		41.6 (4.98)	
Rutkove et al [21]	ALS: 75 (111 consented, and 75 began contributing data)	25 (30 consented and 25 began contributing data)	Baseline characteristics^e^ MND: 65 and controls: 9		Baseline characteristics^e^ MND^b^: 42 and controls: 20		60 (30-80)^f^ and controls: 51 (27-79)^f^		—		—		34 (9-43)^f^	
Rutkove et al [23]	ALS: 72 (113 enrolled and 72 provided data at least once)	—	50		22		60.1 (9.9)		Not disclosed		Not disclosed		36.1 (no SD reported)	
Karas et al [25]	ALS=45	—	29		16		60.1 (10.7)		Symptom onset site: nonbulbar: 31; bulbar: 8; and unknown or not reported: 6		50 (93-281) and unknown or not reported: n=6		36.0 (6.2)	
Holdom et al [26]	MND wrist: 97 and hip^g^: 42	58	MND wrist: 75; MND hip^g^: 31; and controls wrist: 29		MND wrist: 22, MND hip^g^: 11; and controls wrist: 29		MND wrist: 60.69 (IQR 12.55); MND hip^g^: 61.28 (IQR 15.74); and controls wrist: 55.33 (IQR 16.11)		MND subtype: wrist: ALS: 87; progressive muscular atrophy: 1; primary lateral sclerosis: 6; and hip^g^: ALS: 39, progressive muscular atrophy: 3, and primary lateral sclerosis: 0		MND wrist: 21.31 (IQR 13.27) and MND hip^g^: 24.92 (IQR 21.39)		Wrist: 38 (IQR 9) and hip^g^: 38 (IQR 12)	
Johnson et al [27]	ALS: 46 enrolled, 40 met the analysis sample criteria	—	Total: 25; wrist cohort: 12; and ankle cohort: 13		Total: 15; wrist cohort: 8; and ankle cohort: 7		Total: 61.8 (12.0); wrist cohort: 62.9 (13.4); and ankle cohort: 60.6 (10.7)		—		—		Total: 31.4 (8.1); wrist cohort: 31.4 (8.6); and ankle cohort: 31.4 (7.9)	
Gupta et al [28]	ALS: 376	26	ALS: 247 and controls: 14		ALS: 129 and controls: 12		ALS: 57 (21-79)^e^ and 33 (20-67)^f^		First symptoms include: upper limb: 159; lower limb: 164; bulbar symptoms: 75; and respiratory symptoms: 9		22.8 (0-246.3)		41 (14-48)^f^	
van Unnik et al [29]	ALS: 97 (2 cohorts); first cohort^g^: 42) and second cohort: 55	—	Both cohorts: 68; first cohort^g^: 31 and second cohort: 37		Both cohorts: 29; first cohort^g^: 11 and second cohort: 18		Both cohorts: 60.5 (11.1); first cohort^g^:59.9 (11.6); and second cohort: 61 (10.7)		Both cohorts: ALS: 94; progressive muscular atrophy: 3; first cohort^g^: ALS: 39; progressive muscular atrophy: 3; and second cohort ALS: 55		Both cohorts: 22.1 (2.2-217.9; First cohort^g^: 24.9 (6.9-217.9) and second cohort: 18.5 (2.2-93.9)		Both cohorts: 37.9 (6.8); first cohort^g^: 36.3 (8.1); and second cohort: 39.1 (5.3)	
Beswick et al [30]	MND^b^: 10	—	8 8		2		62 (12)		ALS: 5; primary lateral sclerosis: 2; and not disclosed: 3		Survival length: long survivor (>8 years): n=2		40 (6)	
Straczkiewicz et al [31]	ALS: 20^h^	—	12^h^		8i		61.4 (10.6)^h^		Not disclosed^c^		Not disclosed^c^		No baseline reported-estimated baseline total score: 34.4 (30.4-38.3)^h^	

^a^ALSFRS-R: Amyotrophic Lateral Sclerosis Functional Rating Scale-Revised.

^b^MND: motor neuron disease.

^c^Not available.

^d^ALS: amyotrophic lateral sclerosis.

^e^Data taken from Rutkove et al [21] baseline data for the entire group of individuals enrolled.

^f^Data reported as median and range.

^g^As van Eijk et al [24].

^h^As Johnson et al [27] wrist cohort.

#### Follow-Up

Follow-up refers to the overall duration of monitoring completed in a study. Measurement frequency refers to how often participants were invited to wear or use a device (eg, every 3 months), and the measurement duration refers to the period over which measures were recorded on each device deployment (examples are provided in Table 3). The participant follow-up ranged between 12 weeks and 24 months and the measurement frequency was mostly every 1 to 4 months (Table 3). The type of measurement was either periodic (7/12, 58%) or continuous for the duration of the follow-up (3/12, 25%). When the type of measurement was periodic, the duration of each measurement ranged from 1 to 8 consecutive days, with the most common choice being 7 days (3/12, 25%).

**Table 3:** Summary of data collection and data analysis approaches.

	Type and setup of device				Follow-up			Other outcome measures		Data analysis
Study	Body location	Device (type and name)	Sampling frequency (Hz)	Duration of follow-up		Duration and frequency of measurements	Outcome measures		Epochs used in analysis (s)	
van Eijk et al [24]	Right hip (anterior axillary line)	Triaxial accelerometer ActiGraph GT9XLink	30	18 mo		7 consecutive d every 2-3 mo	ALSFRS-R^a^, Hospital Anxiety and Depression Scale, weight, wear time log		10	
Garcia-Gancedo et al [20]	Chest	Triaxial accelerometer Mega faros 180	50	48 wk		3 consecutive d every mo	ALSFRS-R, forced vital capacity, heart rate variability, speech		60	
Kelly et al [22]	Chest	Triaxial accelerometer; Mega Faros 180	50	48 wk		3 consecutive d every mo	ALSFRS-R, forced vital capacity, heart rate variability, speech		60 (based on information from Garcia-Gancedo et al^.^ [20])	
Rutkove et al [21]	Not stated Device designed for wrist	Mi Band R	Not reported	9 mo		Daily for 90 d, then biweekly for 180 d	ALSFRS-R, speech, electrical impedance myography tool, respiratory data, muscle strength, patient-reported experience measures		Not relevant	
Rutkove et al [23]	Not stated Device designed for wrist	Mi Band R	Not reported	9 mo		Daily for 90 d, then biweekly for 180 d	ALSFRS-R, speech, electrical impedance myography tool, respiratory data, muscle strength, patient-reported experience measures		Not relevant	
Karas et al [25]	Not relevant	Personal phone (triaxial accelerometer and GPS)	10 (accelerometer)	Up to 1 y		Continuous cyclical accelerometer 10 s on or off GPS 1 min on or 10 min off	ALSFRS-RSE^b^		60	
Holdom et al [26]	Wrist (nondominant), right hip	Triaxial accelerometer ActiGraph GT9XLink	30	18 mo		Wrist—8 consecutive d every 3-4 months and hip—7 consecutive d every 2-3 mo	ALSFRS-R		10	
Johnson et al [27]	Wrist or ankle	Wrist: triaxial accelerometer ActiGraph Insight Watch, Ankle: biaxial accelerometer Modus StepWatch 4	Wrist: 32 and ankle: 128	6 mo		As much as possible for the duration of the study	ALSFRS-R, ALSFRS-RSE, Rasch-Built Overall ALS^c^ Disability Scale		60	
Gupta et al. [28]	All 4 limbs (wrists and ankles)	Triaxial accelerometer ActiGraph GT3X	30	Minimum of 0.75 y stated		7 d every mo	ALSFRS-R		1	
van Unnik et al [29]	Right hip (anteroaxillary line)	Triaxial accelerometer ActiGraph GT9XLink	30	18-24 mo		3-7 d every 2-3 mo	Survival status, ALSFRS-R (self-administered or physician administered)		10	
Beswick et al [30]	Right wrist and Right ankle	Triaxial accelerometer ActiGraph GT9X	Not disclosed	12 wk		24 h every 2 wk	ALSFRS-R, 6-minute walking test, questionnaires to provide feedback on their experience of wearing devices, standardized series of movements		Not disclosed	
Straczkiewicz et al [31]	Wrist of choice	Triaxial accelerometer ActiGraph, Insight Watch	32	6 mo		Continuously, except for recharging (required every few weeks)	ALSFRS-RSE		60 (for total activity counts)	

^a^ALSFRS-R: Amyotrophic Lateral Sclerosis Functional Rating Scale-Revised.

^b^ALSFRS-RSE: Amyotrophic Lateral Sclerosis Functional Rating Scale self-administered.

^c^ALS: amyotrophic lateral sclerosis.

#### Device Setup

Most studies (8/12, 67%) used commercially available triaxial accelerometers (Table 3). Only 1 (8%) study used a biaxial accelerometer and 1 (8%) study used the participant’s personal smartphone (GPS and triaxial accelerometer). Most (8/12, 67%) studies reported the sampling frequency (Hz) of the device, which ranged from 10 to 128 Hz; however, there was little justification for the choice. The most used frequency was 30 Hz (4/12, 33%). The device wear location varied and included wrist, chest, hip, and ankle. Most studies mounted the device in one place (6/12, 50%), while some studies (n=2, 16%) used 2 cohorts with different wear locations. In total, 2 (16%) studies mounted the devices to several locations, with Gupta et al [28] simultaneously comparing 4 devices (one on each ankle and wrist) per participant and Beswick et al [30] comparing 2 devices (right wrist and right ankle) per participant (Table 3).

#### Other Outcome Measures

All studies used clinician or self-administered ALSFRS-R to track disease progression and used it as a correlation point when assessing the validity of the physical behavior end points. Either a total score or subdomain scores of ALSFRS-R were used (gross motor, fine motor, bulbar, and respiratory). In total, 6 (50%) studies included other outcome measures, such as additional questionnaires (Hospital Anxiety and Depression Scale, Rasch-built Overall ALS Disability Scale, and study specific questionnaires; 4/12, 33%), respiratory data (2/12, 17%), cardiac data (1/12, 8%), speech data (2/12, 17%), muscle strength (1/12, 8%), mobility tests (1/12, 8%), and survival status (1/12, 8%; Table 3). Additional outcome measures were predominantly used as stand-alone measures, although their relationship with the physical behavior end points was assessed in 2 (17%) studies. van Unnik et al [29] found that participants with a lower vertical movement index (Table 4) also experienced a significantly lower probability of survival compared to participants who had a higher vertical movement index during follow-up. Beswick et al [30] assessed the relationship between a mobility test and physical behavior end points from the devices. They found a significant correlation between the distance walked during the 6-minute walking test and the 6-minute walking test total vector magnitude counts from ankle-mounted devices.

**Table 4:** Physical behavior end points used in the studies included in the systematic review^a^.

Study	End points
van Eijk et al [24]	Percentage active—vector magnitude counts >100 counts per minute Metabolic equivalent of task score—average daily metabolic equivalent of task Daily vector magnitude − (vector magnitude average × SD of vector magnitude) Daily A1—variation in vertical axis (y; ie, movement against gravity)
Garcia-Gancedo et al [20]	Activity score algorithms to evaluate how much activity is performed Activity classification algorithms to evaluate what activities are performed
Kelly et al [22]	Daytime, nighttime, and 24-h values for duration of wear time; total activity score Daytime and nighttime values for time and percentage time spent active; time and percentage time spent sedentary (not lying); time and percentage time spent lying; time and percentage time sedentary; maximum activity score; mean maximum activity score; number and average duration of active periods (>1 min) also categorized into 5 categories of activity duration:>1 to ≤2 min, >2 to ≤5 min, >5 to ≤15 min, >15 to ≤30 min, ≤30 min active, Nighttime rest end points: percentage time lying down (at night), number of nighttime movement episodes, number of nighttime movement episodes per h, percentage time nighttime rest efficiency, rest fragmentation index (movement time divided by the number of movement episodes), average duration of movement episodes
Rutkove et al [21]	Steps
Rutkove et al [23]	Steps
Karas et al [25]	Smartphone accelerometer data end points:log (activity index), log (activity index from top one min), walking cadence (steps per s), walking cadence (steps pers) from top one min, log (step count), log (step count from top one min) Smartphone GPS data endpoints: log (distance traveled in kilometers), home time (h)
Holdom et al [26]	Proportion of time active Vector magnitude Variation in axis 1 Variation in axis 2 Variation in axis 3
Johnson et al [27]	Wrist—ActiGraph Vendor-derived measures:light activity (min), moderate activity (min), vigorous activity (min), moderate-vigorous physical activity (min), sedentary (min), nonsedentary (min), locomotion (min), nonlocomotion (min), steps, calories, metabolic equivalent of task, total activity counts, sleep (min), Investigator-derived measures (using actigraphy minute-level activity count):total activity count (24-h activity count sum), log total activity count (logarithmic transformation of total activity counts+1), total log activity count (24-h sum of logarithmic transformation of activity count+1), min spent active (min with activity count >1853), min spent inactive, active to sedentary transition probability, sedentary to active transition probability Ankle—modus Vendor-derived measures: second-level step count data, minute-level step sums, daily level step counts, percentage time in low activity (1-15 steps/minute), percentage time medium activity (16-40 steps/minute), percentage time high activity (41+ steps/minute) activity, mean, median, 95th percentile, peak performance index, and max consecutive (60, 20, 5, and 1 minute) cadences
Gupta et al [28]	The number in the bracket refers to the number of end points per each measure Activity index: activity index mean (1), activity index median (1), activity index mode (1), activity index entropy (1), percentage daytime with low activity index (1), percentage daytime with moderate activity index (1), percentage daytime with high activity index (1), percentage acceleration in single direction (3), Spectral: total power (1) Activity bout: bout acceleration (2), bout jerk (2) Submovement: submovement distance (8), submovement velocity (8), submovement acceleration (8), submovement jerk (8), submovement duration (8), submovement principal component 1 score (6), submovement principal component 2 score (6), submovement principal component 3-5 score (18)
van Unnik et al [29]	Vertical Movement Index—based on movements against gravity
Beswick et al [30]	Total vector magnitude counts Vector magnitude counts from ankle-mounted devices during motor assessments
Straczkiewicz et al [31]	Total daily count of flexions by at least 45°, 90°, and 135° Total daily count of extensions by at least 45°, 90°, and 135° Total daily count of supinations by at least 45°, 90°, and 135° Total daily count of pronations by at least 45°, 90°, and 135° Total daily count of flexions and extensions by at least 45°, 90°, and 135° Total daily count of supinations and pronations by at least 45°, 90°, and 135° Average daily duration of 10 fastest flexions by at least 45°, 90°, and 135° Average daily duration of 10 fastest extensions by at least 45°, 90°, and 135° Average daily duration of 10 fastest supinations by at least 45°, 90°, and 135° Average daily duration of 10 fastest pronations by at least 45°, 90°, and 135° Average daily duration of 10 fastest flexions and extensions by at least 45°, 90°, and 135° Average daily duration of 10 fastest supinations and pronations by at least 45°, 90°, and 135° Total activity counts—a daily (24-h) sum of min–level activity counts

^a^For more details around how these physical behavior end points were derived please refer to the original publications included in the systematic review [20-31].

### Data Analysis

Missing accelerometer data can occur for several reasons, and how this is managed seemed closely related to the device use and wear protocols (eg, charging and overnight wear) and the sampling frequency. Because devices can be removed by the participant, nonwear time must be detected and distinguished from sedentary behavior, and a minimal wear threshold for sample inclusion in analysis must be decided. For instance, Straczkiewicz et al [31] excluded days with <21 hours of cumulative wear time, and van Eijk et al [24], van Unnik et al [29], and Johnson et al [27] excluded samples with <8 hours recording per day from analysis, while Gupta et al [28] excluded samples with <3 hours recording per day. In relation to sampling, Karas et al [25] adopted a smartphone-based acquisition method whereby data collection for accelerometer and GPS cycled between data acquisition periods and periods where no data were acquired (more information can be found in Table 3) to avoid excessive battery drain. Therefore, the sample had missing data a priori, and imputation was performed before analysis.

The raw acceleration signals were commonly processed into epochs (time periods) before analysis. Most studies used 10 seconds (3/12, 25%) or 60 seconds (3/12, 25%) epochs for analysis, while other studies adopted 1-second epochs (1/12, 8%; Table 3). Data were preprocessed and analyzed either via algorithms developed by the research team [20,31] or previously developed and reported algorithms, such as activity index [32] or submovement analysis [33,34]. In addition, some studies [24,26,27,29,30] used proprietary algorithms for data preprocessing provided by device vendors.

Even though researcher-developed algorithms or proprietary software were used for data preprocessing, most physical behavior end points were researcher-derived in line with the specific objectives of the study. Table 4 shows all the physical behavior end point used. The majority focused on daytime behaviors, with only Kelly et al [22] and Johnson et al [27], including any nighttime or sleep-based end points. Due to different sampling frequencies and data preprocessing steps, the end points differed between all studies except 2 (17%) [24,26]. Here, variation of vertical axis, daily vector magnitude, and proportion of time spent active were the defined end points, and were assessed using the same device, sampling frequency, and data preprocessing, although at 2 different wear locations (hip and wrist) [24,26]. Many end points focused on quantifying traditional physical behavior variables, such as the duration, intensity, and frequency of physical activity. In total, 5 (42%) studies explored nontraditional physical behavior end points. Of those, 3 (60%) focused on the end point based on variation in vertical axis developed by van Eijk et al [24], which is based on movement against gravity. Straczkiewicz et al [31] focused on total daily count and average daily duration of upper limb movements, such as flexion, extension, pronation, and supination, among others. Finally, Gupta et al [28] used submovement analysis based on their previously developed algorithms that identified small segments, termed as submovements, within the movement patterns of the wrist, recorded during reaching tasks that had been associated with movement impairments in participants with ataxia [33,34]. In addition, Gupta et al [28] explored the use of artificial intelligence, such as machine learning approaches, for data analysis rather than traditional statistical analysis (eg, linear-mixed effects models), which were used by all studies.

### Reported Research Findings

#### Validating Remote Monitoring of Physical Behavior

The reported research findings consistently demonstrated that physical activity levels decreased longitudinally with MND progression. Moreover, physical behavior end points were associated with total ALSFRS-R score with correlation coefficients (*r*) ranging from 0.31 to 0.78. This was also true for correlation with the gross motor and fine motor domains of ALSFRS-R. In addition, van Unnik et al [29] demonstrated high correlation coefficients of changes in the fine motor domain (Pearson *r*=0.86, 95% CI 0.80-0.90) and gross motor subdomain (Pearson *r*=0.79, 95% CI 0.70–0.85). However, while certain end points (daily vector magnitude and variation in vertical axis; Table 4) resulted in reduced between-patient variability (measured as coefficient of variation), [24] some (eg, average daytime active [min] and percentage daytime active [%]) showed greater variability compared to ALSFRS-R [22].

Device placement influenced reported outcomes. Specifically, wrist-derived outcome measures consistently correlated with functional loss in “fine motor” domain in ALSFRS-R, while measures from hip or ankle-mounted devices were strongly associated with a change in gross motor function [24,26,28] and most recently shown to also correlate with the fine motor domain [29]. In addition, Gupta et al [28] demonstrated that when monitoring all 4 limbs, there was good agreement between right and left limbs for physical behavior submovement outcome measures, with agreement between the left and right ankle stronger (*r*=0.81-0.97) than between the left and right wrists (*r*=0.65-0.82). Moreover, taking the score of a limb with the maximum progression rate produces a motor outcome measure consistent with, but more sensitive than, ALSFRS-R [28]. None of the studies investigated the effects of disease phenotype on physical behavior end points nor the most optimal wear location for each phenotype.

#### Effect of Accelerometer-Derived Outcome Measures on Sample Size Requirements

In total, 5 (42%) studies investigated the effects of using physical behavior end points, including increased measurement frequency, on sample size requirements of hypothetical clinical trials. In total, 4 (33%) studies found that a reduction in sample size would be related to the increased sensitivity of their proposed outcome measures [23,24,28,29]. This was determined either through increasing measurement frequency (daily monitoring) [23], taking the score of a limb with maximum progression rate in a study monitoring all 4 limbs [28], or reduced between-patient variability (and thus increase sensitivity) of end point based on the variation of daily activities [24]. For example, van Eijk et al [24] demonstrated that when recording 7 days of data every 2 to 3 months, end point, such as daily vector magnitude and variation in vertical axis, outperform ALSFRS-R at 9 months and lead to 30% reduction in required sample size at 12 months. Similarly, van Unnik et al [29] demonstrated that for a study with 7-day recordings at monthly intervals with a 6-month follow-up, 50 participants would be required (80% power) to detect differential progression rates of vertical movement index. In addition, van Unnik et al [29] found that if the follow-up duration is increased to 12 months, the sample size can be reduced by 50%. In contrast, Kelly et al [22] found that their physical behavior end point (average daytime active [min] and percentage daytime active [%]) resulted in increased sample size requirement for a hypothetical clinical trial compared to ALSFRS-R total score (500-700 participants for physical activity end points vs 290 participants for ALSFRS-R). This was explained by greater end point variability toward the end of the study compared to ALSFRS-R, possibly due to the relatively small sample size in the reviewed study (n=18) [22].

#### Feasibility of Using Accelerometer Devices in MND

In total, 9 (75%) studies assessed at least 1 or more aspects of feasibility in implementing accelerometer-derived measures of physical behavior in people living with MND. Feasibility was typically assessed via Likert-type, dichotomous or numerical rating scale questionnaires. The assessment of feasibility reported focused on perceptions of participants and did not include input from other individuals, such as clinicians, caregivers, or family members. The overall impression was positive, participants found procedures acceptable [20,30], and reported it improved their sense of control of the disease [23].

Device cost was reported by Gupta et al [28] as US $234-US $433 over the course of the study and by van Unnik et al [29] at US $315 (as of 2021). Garcia-Gancedo et al [20] reported adverse events that occurred during the study, all of which related to skin sensitivity to the adhesive used to secure the device to the participant. In terms of technical challenges, Garcia-Gancedo et al [20] reported 1 electrical failure of a device while it was being charged. Rutkove et al [21] reported challenges regarding manufacturers stopping production of devices used during their study. In the study by Beswick et al [30], no participants reported side effects, nor did they have any concerns about remembering to charge the device or the device interfering with daily activities. They also found that 90% of participants would be happy to wear the devices for longer than 12 weeks, and 70% felt positive about the suggestion that using the device may result in needing to attend fewer clinic appointments.

In total, 2 (17%) studies invited participants to visit the study site, where devices were introduced to participants at setup [20,22]. In contrast, 6 (50%) studies were mostly conducted remotely, with varying levels of details reported regarding whether devices and their instructions were posted to participants and the level of support provided over telephone or videoconference calls [21,23-25,27,31]. The study by van Unnik et al [29] had in-person visits, but for some participants the device was mailed out. Beswick et al [30] carried out in-person visits and used videoconferencing to do the study assessments. Rutkove et al [21] was the only study to report issues related to participants being unable to successfully work the device.

#### Participant Adherence to and Burden of Device Wear

Adherence was assessed in 8 (67%) studies, based on the number of valid wear days (days when the minimum wear threshold of the device was achieved) against the total number of recording days. Overall, adherence was good, ranging from 91.8% to 93% for hip-worn devices [24,29], 92% for chest-worn devices [20], 86% to 95.7% for wrist-worn devices [26,30], and 87.3% for ankle-worn devices [30]. Overall, the number of valid days was higher for wrist-worn devices compared to ankle-worn devices in studies that assessed multiple devices in 1 participant cohort [28,30]. Adherence for chest-mounted monitors reduced longitudinally, from 92% at baseline to 56% at the last measurement, which was explained by physical inability to meet protocol requirements for attaching the device, increased reliance on caregivers to facilitate device use, or decreased willingness to comply with study procedures [20].

Van Eijk et al [24] assessed the wear burden using a Likert rating scale where 0 indicated no burden and 10 indicated high burden. The mean score was 1.3, indicating a low rate of burden for the hip-worn device. Similarly, Garcia-Gancedo et al [20] reported that participants found the chest-mounted device comfortable to wear; however, 24% (6/25) of participants reported symptoms of local skin irritation (itching and skin reaction potentially due to allergy to the adhesive). Beswick et al [30] explored patients’ expectations of wearing devices. They found that 90% of participants thought wearing the devices would be useful for tracking changes in their symptoms.

## Discussion

### Principal Findings

This systematic review investigated current methods, findings, feasibility, acceptability, and implications of remotely monitoring free-living physical behavior in people living with MND. Studies consistently showed that decreased physical activity levels occurred over time, as would be expected with MND progression, and are currently captured by questionnaire-based assessments and clinical observation. However, heterogeneous data collection and analysis procedures were used with little consistency in protocols between studies. Some proposed physical activity end points were found to correlate well with the total ALSFRS-R score and alongside increased monitoring frequency, were shown to provide smaller sample size requirements for hypothetical clinical trials that could be completed within shorter time periods. However, it should be noted that study participants tended to be biased toward slower progressors and those with limb-onset phenotypes (Table 2). In addition, device wear location (eg, upper vs lower limb vs hip) can influence the results, with outcomes derived from wrist-worn devices correlating better with functional loss in the “fine motor” domain of the ALSFRS-R, while hip or lower limb mounted devices were more strongly associated with change in gross motor domain [26,28]. Nevertheless, van Unnik et al [29] were able to evidence a good correlation between hip-worn devices and both gross and fine motor functions. This could have implications for recommendations on optimal strategies for monitoring change across groups presenting with different onset sites. Importantly, studies reported positive feedback on the use of accelerometer devices and good adherence by study participants, although this did decrease longitudinally [20].

### How Are Accelerometer Devices Currently Used to Study MND?

#### Overview

Currently, considerable heterogeneity exists across studies monitoring physical behavior in MND. This is not surprising given the nascent use of these methods in MND research (~5 years), and something that is also seen in other research areas where such methods are much more common [35-38]. Given the rareness of MND (and MND subtypes) and the challenges this presents for accruing large longitudinal datasets, there is value in establishing a consensus approach in MND, and the development of standardized methods of data collection and analysis that would enable harmonization across datasets. This would facilitate data sharing, comparability of findings, and support better phenotyping of MND subtypes. From the results presented here, it seems particularly important to consider developing a consensus across aspects of data acquisition, analysis, and reporting. Therefore, the following subsections summarize key elements of these factors found in the reviewed studies that require further consideration.

#### Data Acquisition

Across the reviewed studies, there were notable differences in total participant follow-up time, frequency, and duration of each measurement period (Table 3). It is important that the total follow-up time should allow observation of clinically relevant changes. The total participant follow-up of 6 to 24 months in the studies captured such changes and sits well within the average MND survival of 2 to 3 years after symptom onset [2]. Studies tended to record data either monthly or every 2 to 3 months, and the latter would coincide with routine clinical assessment or appointment frequency currently recommended by National Institute for Health and Care Excellence [3]. Once meaningful changes in physical behavior is known, remote monitoring could facilitate personalized visit schemes that could reduce the travel burden and cost. However, it is important to note that studies tended to be biased toward more slowly progressing and predominantly limb-onset disease phenotypes (Table 2), and optimal data recording frequency could differ between slower and faster progressors. This concern is not unique to studies on movement, as a bias toward the inclusion of slower progressing patients is well-documented in traditional epidemiological studies. This will, in part, be addressed by the release of data from clinical trials (where inclusion is generally biased toward more rapidly progressing patients) that include measures of movement as part of study outcomes.

To capture accurate information on the participant’s current functional ability, the duration of each measurement period should capture day-to-day variations in behavior [39]. Larger day-to-day variations necessitate longer monitoring periods to be robustly captured; however, day-to-day variations were not reported in any of the reviewed studies. This makes it difficult to identify the optimal duration of recording. Most included studies were recorded over 7 consecutive days (Table 3). This duration is considered adequate to capture most physical behavior variables [39,40] and accounts for variations in social and work activities that occur over a week yet does not exceed the battery life for most commercially available accelerometers [41-43]. However, if future MND specific research establishes that there is little day-to-day variability for MND specific end points, the duration of each measurement could be reduced, which could provide several benefits, including reduced wear burden for participants.

While accelerometers were the most used devices, the method of their attachment and the wear location varied (Table 3). The studies suggest that the wear location has the potential to influence the outcomes [26]. However, the most appropriate location is yet to be determined and will likely depend on the aspect of physical function of main interest (ie, fine vs gross motor skill). Moreover, optimal wear location and the end point most sensitively reflecting physical function may differ across MND presentation and phenotype. Participants living with different MND subtypes were reported to be included in 4 (33%) studies, and ALS site of symptom onset was reported in 3 (25%) studies (Table 2). It is not clear whether people living with different MND subtypes participated in the other studies, and these details were not reported, or whether all participants had the same subtype. Either way, this means the influence of disease-specific factors on the suitability of different physical behavior end point measures has not yet been assessed in detail and represents a gap in current knowledge.

An additional parameter of importance in data acquisition is the device sampling frequency. This varied greatly between the studies (10-128 Hz, Table 3), with limited justification for the frequency used. The major frequency components of human movement are low, occurring up to 20 Hz [44-46]. In gait, most of the energy is contained below 15 Hz; therefore, to conserve 99% of the signal power, the sampling frequency must be a minimum of 30 Hz [44,47], and this was the most used sampling frequency within the reviewed studies (Table 3). However, Khan et al [48] eloquently demonstrated that datasets of different activities (eg, Parkinson disease and walking and physical monitoring) each have different optimal sampling frequency ranges (26-63 Hz). Therefore, the optimal sampling frequency for sensitively detecting changes in physical function in MND, while avoiding battery drain and large storage requirements may warrant further assessment. The MND community will therefore benefit from establishing standardized means of collecting accelerometer data, in a similar manner to that developed by the Mobilise-D consortium [49].

#### Data Analysis: Preprocessing

Reviewing the data analysis approaches used across studies revealed large differences in data preprocessing undertaken to derive physical behavior metrics, which have implications for end point comparison across studies. For example, ActiGraph devices were the most used in reviewed studies (7/10, 70%), and 5 (71%) studies used the manufacturer’s proprietary software (ActiLife) to derive activity counts from the raw data to construct physical behavior end point. However, activity counts can be calculated in different ways (not universal) with a complex relationship between raw data and counts that differs between device models [48]. This will influence the generalizability of results and could also pose challenges when comparing studies that have used different versions of the same software. Therefore, it seems important for researchers to consider the implications of data preprocessing via proprietary algorithms, a point that must be balanced against the availability of technical expertise in the research team. There is likely value in the MND research community working toward the provision of transparent and accessible processing tools (eg, through an Open Science framework, as used to share activity index analysis code) to facilitate replication of findings and standardization across study sites.

**Figure 2:**
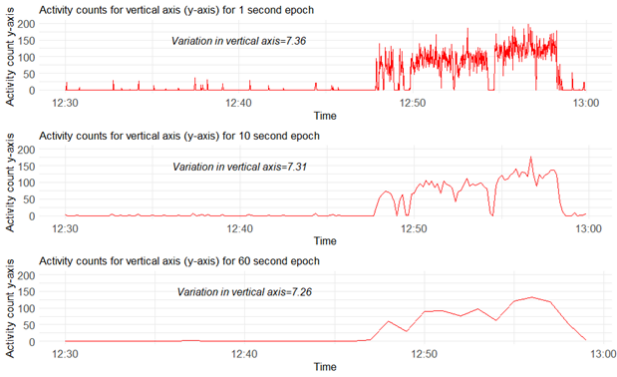
Graphical representation of 30-minute segment data from a healthy individual in free-living conditions wearing a right anterior thigh-mounted triaxial GENEActiv accelerometer with a sampling frequency at 100 Hz. The figure demonstrates standardized activity counts of y-axis at 1-, 10-, and 60-second epochs. Variation in y-axis was calculated as per van Eijk et al [24].

A further important feature of data analysis that warrants consideration is the reduction of data into epochs. This approach of smoothing data was routinely used across studies, with 10- or 60-seconds epochs commonly used. Despite this being routine practice, there is a lack of consensus on the most appropriate epoch length for specific, measurable outcomes. Because epoch length determines data resolution (Figure 2), the choice should likely be guided by the behavior of interest. End points relating to subtle movement variations, moving from sitting to standing or reaching and grasping, may warrant shorter epoch lengths (eg, 1 second). In contrast, end points relating to physical behaviors, such as walking, may warrant longer epoch lengths (eg, ≥10 seconds). Therefore, future research should consider and justify the choices of data smoothing to balance the difference in information against the feasibility and demands of processing large quantities of uncompressed data.

#### Data Analysis: Physical Behavior End Points

Most (7/12, 58%) of the studies only reported traditional physical behavior end points, which typically summarize the overall frequency, intensity, and duration of physical activity. In addition, changes to end points specific to sedentary behavior patterns could also be explored [37]. However, these measures represent a broad overview of physical behavior but do not provide information on movement characteristics. In addition, it seems inevitable that these measures will decline with MND progression, and some (eg, average daytime active [min] and percentage daytime active [%]) even lead to greater data variability compared to the ALSFRS-R [22]. Thus, when used in isolation, some outputs may not be optimally suited as clinical end points. In contrast, end points that go beyond measuring the volume of physical behavior and focus more on movement characteristics, quality, or complexity (ie, *how* people move rather than *how much* they move) may have greater potential value. Examples of such measures include the daily vector magnitude [24] and submovement analysis [28], with vector magnitude having been shown to reduce between-patient variability compared to traditional measures, such as the percentage of time spent active and thereby increasing sensitivity to disease progression [24,26].

#### Reporting of Physical Behavior Studies in MND

The growing interest in studying free-living physical behavior end points in MND means there is value to ensuring transparent and clear reporting. This would facilitate the identification of problems in conducting studies as well as the clarity and quality of reporting and hence accelerate consensus around optimal study designs. For example, a clear representation of participants at each stage of the study, potentially in the form of a flowchart, would allow a clear reflection of longitudinal attrition rates and their causes. In addition, there should be justification for design choice, including a clear description of the type of follow-up, accelerometer location, method of attachment, and sampling frequency. Consideration should also be made of the requirements for reporting data preprocessing, as this is something that differed significantly between studies. Consequently, work to develop standard reporting guidelines would be particularly timely and valuable to the MND community. Examples of such efforts in other fields that could provide a useful foundation for the MND community include the recommendations for assessing and reporting human joint kinematics using inertial measurement units recently reported in the biomechanics community but has yet to demonstrate its impact [50].

### Perspectives on the Feasibility of Monitoring Physical Behavior in MND

#### Overview

When recorded, reviewed studies consistently found that participants had positive attitudes toward remote monitoring of physical behavior, excellent use, and reported a low rate of burden. Despite limited evidence specific to accelerometry, other studies of telehealth tools in MND also found that people living with MND are accepting toward using remote monitoring approaches [12,51]. However, it is clear that participants face challenges using digital devices over the course of study. For example, the physical challenge of removing and reattaching a device may, coupled with the progression of MND, influence adherence [20]. Gupta et al [28] and Beswick et al [30] have also shown that the amount of use differs between wear locations. However, there was very limited information on factors that reduced use (including consideration of family member or caregiver burden) provided in the articles included. This restricts the evidence base on which future study protocols can be optimized to maintain participant involvement as the disease progresses and avoid missing data. Further research is needed, and we are currently working to expand our knowledge of the influencing factors.

#### Future Considerations of Physical Behavior Monitoring in MND

To support the development of a consensus approach for the quantification of physical function from digital devices in MND and enhance the opportunity for data harmonization, we present currently unanswered questions and recommendations for future research in this area (Table 5). None of the reviewed studies evaluated the use of devices for clinical care, and research has not explored the implications of remotely monitoring physical behavior on clinical care. Further research is warranted, and it is likely that further considerations will need to be made to mature and translate the technology for clinical practice. The focus of this review was to explore monitoring of free-living physical behaviors, where individuals are completing tasks of their choice. It should be noted that digital devices can also be used in clinics or home-based assessments of defined functional tests, such as sit-to-stand tests [14,52]. These approaches are especially useful when standardizing data acquisition, analysis, and reporting protocols.

**Table 5:** Current unanswered questions in motor neuron disease (MND) actigraphy research and future recommendations, ranked in terms of perceived importance.

Question	What is known so far?	Recommendation
User-related: are these devices and procedures feasible for use by people living with MND?	Adherence is good, and participants largely thought the devices were acceptable and reported a low burden of use.	Research to explore people’s lived experience of using the devices. Qualitative research methods will enable in-depth exploration of feasibility and allow identification of barriers and facilitators to using digital technologies.
User-related: are the devices and procedures feasible for family members, caregivers, and health care professionals?	Research has not comprehensively explored the experiences of individuals using the devices and procedures.	Research to explore experiences and perceptions of these individuals. Qualitative research methods will be helpful in identifying barriers and facilitators.
Clinical practice-related: is physical behavior related to other symptoms of MND?	Physical behavior is only a small part of MND, and no research has investigated relationships with other relevant disease domains.	Research to explore whether physical behavior is related to other objective measure areas (eg, respiratory function and muscle strength).
Clinical practice-related: are physical behavior end points more sensitive measures of disease-related change in physical function than the ALSFRS-R?	The evidence is inconclusive. Some studies have found that accelerometry data have greater variability than the ALSFRS-R^a^, while others found less variability than the ALSFRS-R.	Research to quantify variability in physical behavior end points relative to that in ALSFRS-R. Consideration of effects of different MND phenotypes on measurement variability will be required here, as well as estimation of clinically meaningful effect size.
Methods-related: what is the optimum follow-up design to capture changes in physical behavior?	There is no consensus on the duration of follow-up, frequency of measurement, or duration of measurement. A measurement period of 7 days can account for potential day-to-day variation in physical behavior.	Research to identify the optimum durations of follow-up, frequency of measurement, and length of measurement. Using qualitative methods to explore people’s experiences of this will also contribute to our knowledge of what is feasible for patients and health care professionals.
Methods-related: what is the most optimal wear location to capture and predict changes in physical behavior with MND progression?	The wrist location correlated better with the ALSFRS-R fine motor domain and lower limb placement (hip or ankle) correlated better with the gross motor domain. Physical behavior end points may need to vary based on device wear location.	Research to identify optimal wear locations, including consideration of impacts on use of other devices or collection of additional data (eg, pulse oximetry). Studies should consider ease of use and participant burden and impacts of their evolution with disease progression.
Methods-related: is there an optimum device location and outcome measure for each MND phenotype?	Research has not investigated whether there are differences in outcome measures between MND phenotypes.	Research to explore inertial measurement unit performance, optimum wear location, and physical behavior end points across MND phenotypes.
Clinical practice-related: does monitoring physical behavior offer a cost-effective means of assessing change in physical function?	Research has not explored the cost implications or economics associated with using physical behavior end points.	Research evaluating the cost-effectiveness of using physical behavior end points in both clinical trials and in care is required.
Clinical practice-related: do physical behavior end points provide information that is clinically relevant or related to clinical milestones?	Research has not explored the impact of physical behavior end points on clinical decision-making, nor relationships to milestones (eg, loss of ambulation or care dependency).	Research to explore how using devices will impact clinical decision-making. Future studies should aim to establish minimum clinically important differences and minimal detectable change values for commonly used physical behavior end points. Qualitative research methods will be helpful for exploring this in depth.

^c^ALSFRS-R: Amyotrophic Lateral Sclerosis Functional Rating Scale-Revised.

### Conclusions

Remote monitoring of free-living physical behavior in people living with MND is in its infancy but has exciting potential to quantify physical function in MND. Most research to date has aimed to describe changes in physical behavior associated with MND progression or identify physical behavior end points that are more sensitive than the ALSFRS-R and may be used in clinical trials to decrease sample sizes. Exploration of feasibility in all end users is necessary as this will help to translate the technology into clinical practice and will also help guide the design of future studies through cocreation with patient and caregiver involvement.

It is essential to develop a consensus statement within the MND community, working toward agreed and standardized methods for data collection, analysis, and reporting. The unanswered questions and recommendations for future research (Table 5) offer a foundation from which such efforts can begin. While aspects relating to study design will take longer to resolve, agreement on standards for reporting should be achievable in the shorter term. This is important in facilitating future data harmonization across cohorts, study replication, and standardizing collection and analysis procedures.

## Data Availability

Data sharing is not applicable to this paper as no datasets were generated or analyzed during this study.

## Appendix

Multimedia Appendix 1 PRISMA 2020 checklist.

Multimedia Appendix 2 Data extraction tool used to carry out the systematic review.

## Abbreviations

ALS: amyotrophic lateral sclerosis
ALSFRS-R: Amyotrophic Lateral Sclerosis Functional Rating Scale-Revised
MND: motor neuron disease
PRISMA: Preferred Reporting Items for Systematic Reviews and Meta-Analyses
STROBE: Strengthening the Reporting of Observational Studies in Epidemiology
