# Supplementary material for: The Use of Digital Devices to Monitor Physical Behavior in Motor Neuron Disease: Systematic Review
Source: J Med Internet Res. 2025 Apr 17;27:e68479. doi: 10.2196/68479 (PMC12046257; doi:10.2196/68479)
Supplement: Multimedia Appendix 2 [file jmir_v27i1e68479_app2.docx]

**Multimedia Appendix 2: Data extraction tool used to carry out the systematic review**

| **Subsection** | **What was extracted** | **Additional information** |
| --- | --- | --- |
| **General info** | Title | What was the title of the study? |
|  | Author and Year | What is the name of the lead author?  What is the year of publication?  (e.g. Name et al. 2009) |
|  | DOI | N/A |
|  | Setting (country) | What country was the study based in? |
| **Method** | Aim/Objectives of the study | What were the stated aims and objectives of the study? |
|  | Design | N/A |
|  | Duration of study | What was the length of follow up? |
|  | Frequency of measurements (eg. 2 monthly) | How frequently were participants measured (measurement interval)? |
|  | Duration of each measurement | How long did each measurement take? |
|  | Device used | What device was used and what was the name of device? (e.g Triaxial accelerometer ActiGraph GT9XLink) |
|  | Body location | Which location of the body was the device attached to? |
|  | Method of attachment | How was the device attached? |
|  | Return of devices (if applicable) | How was the device returned to the research team? |
|  | Charging | Did the device require charging by the participants? If yes, how frequently? |
|  | Sampling frequency (eg. 30Hz) | What was the sampling frequency of the device? (e.g. 30Hz) |
|  | Device cost (if reported) | What was the cost of the device? |
|  | Physical behaviour outcome measures | What were the physical behaviour outcome measures/endpoints? |
|  | Other outcome measures | What were the other outcome measures used? |
|  | Sample power (if reported) | Was the sample power calculated? If yes, what was it? |
| **Participants** | MND/ALS participants (at baseline) | What was the number of MND participants at baseline? |
|  | Controls (at baseline) | Did the study have controls? If yes, how many at baseline? |
|  | MND subtype | Was MND subtype or ALS phenotype at onset assessed? |
|  | ALSFRS-R scores (at baseline) | What was ALSFRS-R baseline score? |
|  | Age (at baseline) | What was the age at baseline? (MND and controls)? |
|  | Sex (at baseline) | What was the sex ratio at baseline? |
| **Statistical analysis** | Management of missing data | How was missing data managed? |
|  | Type of statistical analysis | What type of statistical analysis was used? E.g. Linear mixed models, etc.) |
| **Results** | Main Findings | What were the main findings of the study? |
|  | Other findings (if applicable) | Were there any additional findings? |
|  | Participant adherence | What was participant adherence to wearing the device? |
|  | Participant number longitudinally | How many MND/ALS participants were followed-up longitudinally + timepoint? (e.g. 27 participants at 9 months) |
|  | Rate of burden of wearing device | What was the rate of burden of wearing the device? |
| **Discussion** | Applicability of the study | How was this study applicable to the research area? |
|  | Limitations | What were the limitations of the study? |
|  | Future suggestions | What future research suggestions did the study provide? |
| **Conclusion** | Key conclusion points by authors | What were the key conclusions of the study? |
